# 

**Published:** 1875-02

**Authors:** 


### CONTENTS:

ORIGINAL COMMUNICATIONS:

ART. I. Medical Expert and Medical Evidence—*continued.* By M. A. McClelland, M.D. .... 81

ART. II. Ether versus Chloroform. By E. W. Lee, M.D. .... 108

PROGRESS IN MEDICAL SCIENCES:

ART. I. Progress of Surgery. By Jno. E. Owens, M.D. .... 119

ART. II. Progress of Obstetrics. By E. Warren Sawyer, M.D. .... 126

REPORTS OF SOCIETIES:

Chicago Med. Society—Paper on the Obstetric Forceps, by Philip Adolphus, M.D. .... 130

Central Ill. Med. Society—Report on "Minor Operations in Surgery," by J. L. White, M.D. .... 141

CLINICS:

Ill. Charitable Eye and Ear Infirmary—Lecture by E. L. Holmes, M.D. .... 148

Rush Med. College—Clinic by Prof. Gunn .... 152

EDITORS' BOOK TABLE .... 154

MORTALITY STATISTICS, Chicago .... 158

